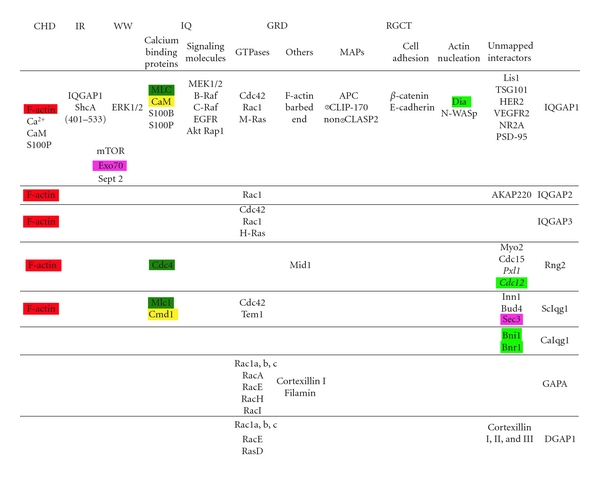# IQGAP Family Members in Yeast, *Dictyostelium*, and Mammalian Cells

**DOI:** 10.1155/2012/894817

**Published:** 2012-02-21

**Authors:** Katie B. Shannon

**Affiliations:** Department of Biological Sciences, Missouri University of Science and Technology, Rolla, MO 65409, USA

## Abstract

IQGAPs are a family of scaffolding proteins with multiple domains, named for the IQ motifs and GTPase activating protein (GAP) related domains. Despite their GAP homology, IQGAP proteins act as effectors for GTP-bound GTPases of the Ras superfamily and do not stimulate GTP hydrolysis. IQGAPs are found in eukaryotic cells from yeast to human, and localize to actin-containing structures such as lamellipodia, membrane ruffles, cell-cell adhesions, phagocytic cups, and the actomyosin ring formed during cytokinesis. Mammalian IQGAPs also act as scaffolds for signaling pathways. IQGAPs perform their myriad functions through association with a large number of proteins including filamentous actin (F-actin), GTPases, calcium-binding proteins, microtubule binding proteins, kinases, and receptors. The focus of this paper is on recent studies describing new binding partners, mechanisms of regulation, and biochemical and physiological functions of IQGAPs in yeast, amoeba, and mammalian cells.

## 1. Introduction

**Figure 1:**
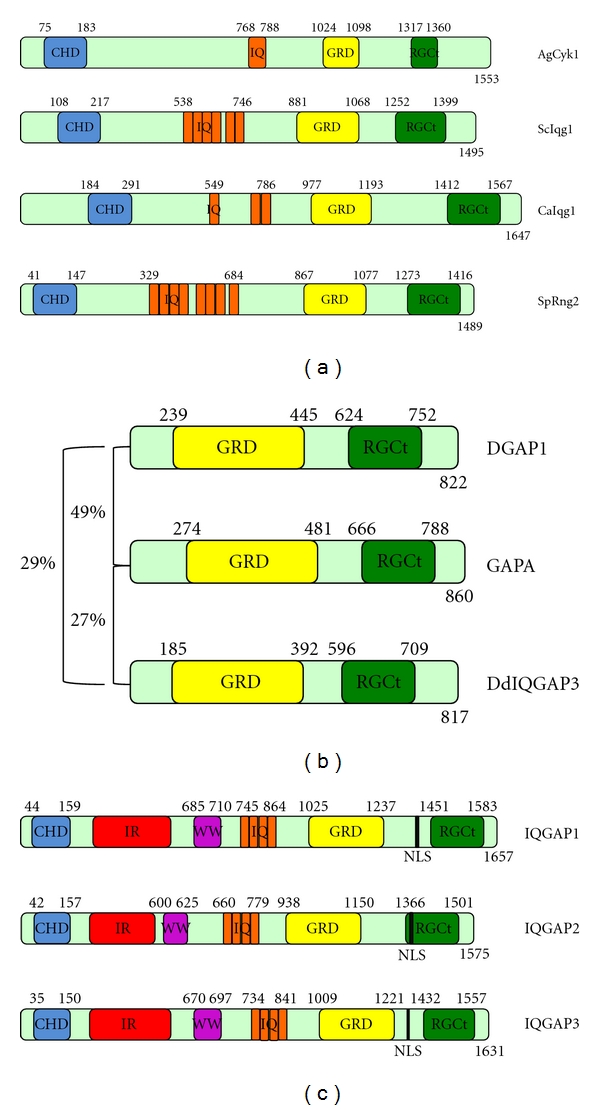
IQGAP domain structure in yeasts, *Dictyostelium discoideum,* and mammalian cells. Domain boundaries were determined by MotifScan using Prosite and Pfam sources [2]. (a) IQGAP family members from yeast. The most related members are AgCyk1 31% identical to ScIqg1, CaIqg1 20% identical to ScIqg1 and 24% identical to SpRng2. Note that additional IQ motifs were identified in CaIqg1 and ScIqg1 by visual scanning [3]. (b) IQGAP family members characterized in *Dictyostelium discoideum*. Percent identify at the amino acid level is shown for each pair. (c) Human IQGAP family members. CHD-calponin homology domain, IR-internal repeats (coiled-coil region), WW-tryptophan containing repeats, IQ-isoleucine and glutamine rich repeats, GRD-GAP-related domain, RGCt-Ras GAP C-terminus homology domain NLS, nuclear localization sequence. Note that not all members of the family contain each domain found in IQGAP1. Although WW domains of other proteins have been shown to bind to proline-rich proteins [4], no such interactions have yet been described for IQGAPs.

IQGAP family proteins are large, multidomain proteins conserved from yeast to human cells. Through multiple binding partners, IQGAPs regulate numerous cellular processes including adhesion, motility, signaling, exocytosis, and cytokinesis. IQGAP1, identified in 1994, is the best-studied family member. IQGAP1 contains a calponin homology domain (CHD) in the N-terminus that binds F-actin, internal repeats (IR) that form a coiled-coil region required for dimerization, a tryptophan repeat motif (WW) of unknown function, IQ domains that bind calcium-binding proteins, a GAP-related domain (GRD) that interacts with Cdc42 and Rac1, and a Ras-GAP C-terminus (RGCt) homology domain (Figure 1) [1]. Homologs of IQGAP1 in yeasts and *Dictyostelium* contain some, but not all, of these domains (Figure 1).

IQGAPs function in cytoskeletal rearrangements downstream of Rho family GTPases and signaling pathways downstream of multiple receptors. Human IQGAP proteins function in cancer cell metastasis, and altered expression of IQGAP1 and IQGAP2 is implicated in tumorigenesis (reviewed in [1, 5]). In this paper, recent studies of IQGAPs in cytokinesis, motility, and signaling will be considered, beginning with yeast IQGAPs, following with studies in the amoeba *Dictyostelium discoideum*, and concluding with mammalian IQGAPs. Conservation of regulation, binding partners, and function will be discussed.

## 2. IQGAPs in Yeast Cells

### 2.1. *Schizosaccharomyces pombe* Rng2

*Schizosaccharomyces pombe* has a single IQGAP family member, Rng2, which is required for cytokinesis [6]. Recent studies in fission yeast have provided details of the molecular function of Rng2 in recruitment of F-actin and other proteins to the actomyosin ring. The formation of the actomyosin ring in this yeast normally begins with the formation of small foci of cytokinesis proteins called nodes. These nodes then coalesce, likely through myosin II pulling on actin filaments nucleated from nearby nodes, leading to formation of the actomyosin ring [7]. A protein called Mid1 specifies the position of the division site and is required for node assembly [8, 9]. However, fission yeast can form contractile rings in the absence of Mid1 and nodes, revealing the presence of a node-independent mechanism of ring formation [10]. Rng2 is required for actin ring formation by both node dependent and independent mechanisms [6].

 Rng2 is important for the recruitment of other cytokinesis proteins to cortical nodes. Rng2 was previously shown to be a node component [9], and a recent study examining the temporal order of node localization demonstrated that Rng2 and its binding partner Cdc4 (also a light chain for myosin II) are the earliest proteins to localize to the nodes after Mid1 [11]. The localization of Rng2 and Cdc4 to nodes is interdependent, and may be mediated by binding of Rng2 to Mid1 [11–13]. Localization of Rng2 to nodes depends on the C-terminus, which contains the GRD and RGCt domains, and this region of Rng2 interacts with Mid1 *in vitro* [13, 14]. In cells with a newly isolated temperature-sensitive Rng2 allele, *rng2-M1*, Mid1 nodes form, but do not coalesce into a ring and instead seem to drift away from the cell middle [12]. Thus, Rng2 appears to have a role in retaining the medial localization of Mid1. Once recruited to nodes, Rng2 may stabilize Mid1 and/or create a positive feedback loop that recruits more Mid1. This model is supported by data showing that the amount of Mid1 in nodes increases around the time that Rng2 and Cdc4 arrive at nodes, and cells with a deletion of the *RNG2* gene (*rng2Δ*) or a temperature-sensitive allele of *RNG2* (*rng2-D5*) have 25% less Mid1 in their nodes [11]. In addition, Rng2 is required to recruit type II myosin and its regulatory light chain (Myo2 and Rlc1) to nodes [11, 12]. Rng2 and Myo2 coimmunoprecipitate, so the recruitment may be due to direct binding of the proteins [11]. Consistent with its role as a scaffold protein in the node, fluorescence recovery after photobleaching (FRAP) analysis demonstrated that Rng2 is relatively stable (*t*_1/2_ = 1.77 min), while Myo2 and Rlc1 are more dynamic (*t*_1/2_ = 0.51 min) [11]. Thus, Rng2 is required to recruit and stabilize proteins at the node essential for formation of the actomyosin ring. Rng2 has also recently been shown to interact with Cdc15, the fission yeast PCH protein that promotes F-actin assembly in the ring, but as Cdc15 does not require Rng2 to localize to nodes, the function of this interaction is unknown [11, 15].

 Two recent studies identified a fission yeast paxillin homolog, Pxl1, and showed that it was a nonessential component of the contractile ring [16, 17]. Paxillins are multidomain scaffolding proteins that are essential components of focal adhesions in mammalian cells. Fission yeast cells that lack Pxl1 (*pxlΔ*) have cytokinesis defects including disorganized actomyosin rings and delayed contraction [16, 17]. Pxl1 interacts with and negatively regulates Rho1 [17]. Both studies showed genetic interactions between Pxl1 and Rng2. In one case, *pxlΔ* was shown to be lethal in combination with *rng2-D5* [17]. In the other study, the double *pxlΔ rng2-Δ5 *strain was viable, but exhibited an extreme cell separation phenotype at 24°C, indicating that cytokinesis was defective [16]. Therefore, these studies suggest that either Pxl1 interacts with Rng2 to promote proper actomyosin ring contraction or these proteins function in parallel pathways that are important for cytokinesis.

**Figure 2:**
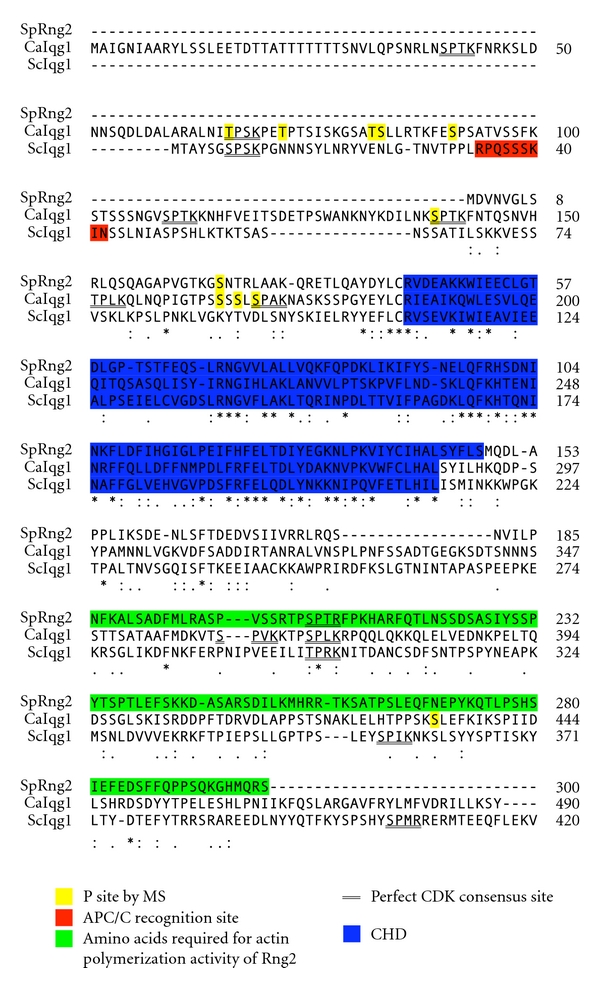
Amino acid sequence alignment of the N-terminus from *S. pombe* (SpRng2), *C. albicans* (CaIqg1) and *S. cerevisiae* (ScIqg1). Amino acids 1–300 of Rng2, which were identified as important for F-actin nucleation activity of Rng2 *in vitro*, were aligned with the N-terminal 490 amino acids of CaIqg1 and 420 amino acids of ScIqg1 using Clustal 2.1 multiple sequence alignment default settings at http://www.ebi.ac.uk/Tools/msa/clustalw2/. Amino acids highlighted in yellow were identified as phosphorylation sites by mass spec analysis, doubly underlined amino acids represent consensus CDK sites (S/TPxK), amino acids highlighted blue are the CHD, ScIqg1 amino acids highlighted in red are the APC/C recognition site, and Rng2 amino acids highlighted green are those identified as being important for F-actin nucleation activity.

 A recent study of the biochemical activity of Rng2 provides insight into the mechanism by which Rng2 functions to recruit and organize F-actin into the ring. A portion of Rng2 that contains the CHD was shown to cross-link and bundle actin filaments [14]. An additional 111 amino acids following the CHD fragment enhance the binding and bundling activity but do not on their own interact with F-actin (Figure 2) [14]. Interestingly, the CHD plus these additional amino acids (Rng2Ns) acted as a nucleation factor in pyrene actin assembly assays [14]. Alignment of the Rng2Ns domain (aa 1–300) with *C. albicans* and *S. cerevisiae* orthologs shows that this region is conserved (Figure 2). Therefore, the actin nucleation activity of IQGAPs may be conserved among yeast cells. Rng2 is the first reported IQGAP family member that acts to promote actin filament assembly *in vitro*. Rng2 may cooperate with Cdc12 to generate contractile ring actin filaments. This hypothesis is supported by the synthetic sick phenotype that results from combining the temperature-sensitive alleles *rng2-D5* and *cdc12-112* [6]. Cdc12 is a member of the formin family, a group of actin nucleating proteins required for cytokinesis in many organisms. The interaction between IQGAPs and formins may be conserved from yeast to human cells (see Section 8).

In summary, Rng2 has multiple roles throughout the assembly and contraction of the actomyosin ring. Rng2 is an early component of nodes that are the precursor to the contractile ring. It may act as a scaffold, stabilizing and increasing the amount of Mid1 and recruiting myosin and its light chain. Since the assembly of cytokinesis proteins into nodes is regulated by the Polo kinase Plo1, it will be interesting to determine if phosphorylation of Mid1 by Plo1 regulates binding to Rng2 [13]. Rng2 is essential for recruitment of F-actin to the ring in both node dependent and independent pathways, but the mechanism by which Rng2 leads to F-actin recruitment to the ring is unresolved. Rng2 may act *in vivo* as a nucleator synergistically with Cdc12 or function to bundle and organize F-actin assembled by Cdc12.

### 2.2. *Saccharomyces cerevisiae* Iqg1/Cyk1

Budding yeast also has a single IQGAP family member, Iqg1/Cyk1, which is required for recruiting actin to the contractile ring though the CHD [18–20]. Budding yeast have a single type II myosin, Myo1, which localizes to the site of cell division (the bud neck) early in G1, and contracts in late mitosis coincident with the reorganization of the septin collar into a double-ring structure [21]. A recent study concluded that Iqg1 is required for Myo1 localization to the contractile ring during cytokinesis, as Myo1-GFP leaves the bud neck at the time of the septin ring split in *iqg1Δ* cells [22]. This may be analogous to *S. pombe* Rng2 recruitment of Myo2 to nodes, but no direct binding was observed between Iqg1 and Myo1 [22]. Another interpretation of these results is that the Myo1-GFP ring is lost from the bud neck due to disassembly in the absence of contraction, as normally disassembly and contraction of Myo1 are coordinated [23]. Previous experiments found persistent Myo1-GFP localization at the bud neck in cells lacking Iqg1 [20, 23]. Further studies are needed to determine whether or not Iqg1 has a direct role in localizing Myo1 to the actomyosin ring during late mitosis.

 Budding yeast can survive without Myo1, dividing by formation of an aberrant septum [24, 25]. Yeasts have both a plasma membrane and a cell wall, and cytokinesis is accompanied by new cell wall deposition resulting in a septum between the two cells. Since Iqg1 is essential but myosin II is not, Iqg1 must participate in other processes besides actomyosin ring assembly and contraction. A temperature-sensitive allele of *IQG1*, *iqg1-1*, is viable at 32°C, even though it fails to form actin rings [26]. Combination of the *iqg1-1* allele with mutations in mitotic exit network (MEN) genes led to lethality at 32°C, demonstrating a synergistic effect [26]. Examination of F-actin in the mutants showed that while single *iqg1-1 *or MEN mutants could repolarize actin to the bud neck after mitosis, this process fails in the double mutant cells [26]. Therefore, the activity of Iqg1 and the MEN is required for reorganization of F-actin subsequent to cytokinesis, which may be important for septum formation through secretion and/or cell polarization. Iqg1 has been shown to interact with the exocyst component Sec3, and *iqg1Δ sec3Δ* double mutants are defective in polarity and bud site selection and have aberrant septal structures [27]. These data suggest that Iqg1 functions along with the MEN and Sec3 for proper repolarization of F-actin and secretion for cell wall assembly following cytokinesis.

 Recently, levels of Iqg1 were shown to increase during mitosis and rapidly drop as cells exit mitosis [25]. Iqg1 is a target of the anaphase promoting complex/cyclosome (APC/C), which regulates late mitotic events by targeting proteins for destruction. Iqg1 is ubiquitinated *in vitro*, and Iqg1 levels are stable in APC/C mutants [25]. Although Iqg1 contains a KEN box and a GKEN, which are known APC/C recognition motifs and would be expected to mediate the ubiquitin-mediated degradation; in fact, the sequence RxxxxxxxN between amino acids 34–42 was shown to be required for degradation of Iqg1 (Figure 2) [25]. Iqg1 lacking the first 42 amino acids, *Iqg1Δ42*, is more stable, and able to rescue *myo1Δ* lethality and cytokinesis defects [25]. *Iqg1Δ42* prevents normal disassembly of the Myo1 ring after contraction, and leads to mild primary septum formation defects [23]. Therefore, the APC/C regulates actomyosin ring disassembly, which is important for proper septum formation, partly through regulation of Iqg1 levels at the end of mitosis [23, 25]. However, the region identified as essential for regulation of ScIqg1 by APC/C does not appear to be conserved among yeast IQGAPs (Figure 2).

### 2.3. Filamentous Fungi *Candida albicans* and *Ashbya gossypii* IQGAPs

Recently, the yeast *C. albicans* IQGAP family member was identified by homology and shown to be required for actin ring formation and cytokinesis like its orthologs in budding and fission yeast [3]. The CaIqg1 has 8 perfect and 13 minimal consensus sites for phosphorylation by cyclin-dependant kinase (CDK), all but 3 of which are in the N-terminus flanking the CHD (perfect consensus sites shown in Figure 2). Iqg1 was shown to be a substrate of the CDK Cdc28 *in vitro*, and mutation of the first 15 consensus Ser or Thr residues to Ala (*Iqg1-15A*) or Asp (*Iqg1-15E*) resulted in cytokinesis defects [3]. Phosphorylation by CDK may regulate the level of Iqg1, as Iqg1-15A was more stable and exhibited premature actin ring assembly, while Iqg1-15E was less stable and showed more severe cytokinesis defects [3]. Iqg1 also interacted with the formins Bni1 and Bnr1 by coimmunoprecipitation, and the interactions were disrupted by the Iqg1-I5A mutation [3]. These data suggest that the interaction between IQGAPs and formins is conserved from yeast to human cells, and that it may be regulated by phosphorylation of IQGAP [3, 28]. While CaIqg1 is a CDK target and ScIqg1 contains numerous CDK consensus sites, Rng2 has only a single perfect CDK site in its N terminus (Figure 2). Therefore it remains to be seen whether regulation of yeast IQGAPs by CDK is conserved.

 The filamentous fungus *Ashbya gossypii* has an IQGAP homolog AgCyk1 that is 30% identical to ScIqg1 [29]. Like IQGAPs in other yeasts, AgCyk1 is required for formation of actin rings [29]. A recent study in *A. gossypii* showed that it also contains a PCH family protein, Hof1 that functions in cytokinesis [30]. In this yeast, proteins involved in cytokinesis including Hof1 and Cyk1 assemble first into a collar of bars, then shorten and transition into a ring [30]. Analysis of Cyk1-YPF in *hof1Δ* cells showed that Hof1 is required for efficient targeting of Cyk1 to the site of septation [30]. Cyk1 is required for the Hof1 transition from bar to ring structures [30]. Therefore, these authors were able to establish a series of stages resulting in cytokinesis and septation in *A. gossypii*, and showed that IQGAP in this yeast is required for development of the cytokinetic ring from the precursor bar structure [30]. These bars may be analogous to the nodes in *S. pombe*, as both assemble prior to rings in an IQGAP-dependent manner and coalesce into F-actin containing rings.

## 3. *Dictyostelium discoideum* IQGAPs

The amoeba *Dictyostelium discoideum* has four IQGAP family members based on analysis of the sequenced genome [31]. Three of these have been characterized in the literature, DGAP1/DdIQGAP1, GAPA/DdIQGAP2, and DdIQGAP3, all of which contain a GRD and RGCt, but lack the N-terminal domains found in mammalian IQGAPs [31, 32] (Figure 1). Although there are conserved IQ and LQ residues in the N-terminus of the three characterized *Dicty* IQGAPs, they lack the consensus L/IQXXXRXXXXR [3, 18]. Therefore, whether or not these are functional IQ motifs awaits determination of interactions between IQGAP family members and calmodulin-like proteins in *Dictyostelium*. DGAP1 and GAPA are 49% identical to each other while DdIQGAP3 is 29% and 27% identical to DGAP1 and GAPA, respectively (Figure 1).

 A recent study examined the role of three *Dictyostelium* IQGAPs in chemotaxis [33]. Two of these proteins, DGAP1 and GAPA, had previously been shown to be required for cytokinesis [34, 35]. The authors constructed and characterized a null mutant of a third *Dicty* IQGAP called DdIQGAP3 encoded by the *iqgC* gene (DdIQGAP3 appears to correspond to DDB0233055) [33]. Single mutants showed little change in directional movement, but loss of GAPA in combination with either DGAP1 or DdIQGAP3 led to chemotaxis defects, suggesting that GAPA is important for directionality and that DGAP1 and DdIQGAP3 have overlapping functions [33]. Signaling pathways activated by chemoattractant lead to activation of Ras and its downstream effectors phosphatidylinositol 3-kinase (PI3K) and AKT. Loss of both DGAP1 and GAPA leads to prolonged activation of AKT after stimulation, but no change in Ras activation [33]. This effect may be mediated by binding to F-actin, as it is phenocopied by lack of the IQGAP binding partners cortexillin I and II, which contain actin binding domains [33]. It is interesting that *Dictyostelium* IQGAPs play a role in regulation of AKT signaling, as a role for mammalian IQGAPs in mediating AKT signaling has also recently been described (see Section 5.8).

 A recent report focused on GAPA showed that it localizes to the furrow during cell division, and that it forms oligomers, likely trimers, using the GRD domain [36]. Mammalian IQGAPs are homodimeric, and it is surprising that GAPA can form oligomers even though it lacks the coiled-coil repeats (IR) that mediate IQGAP1 self-association (Figure 1). GAPA null cells had lower levels of F-actin while cells overexpressing GAPA had increased F-actin, suggesting that GAPA helps to regulate the actin monomer/filament ratio in these cells [36]. Using coimmunoprecipitation, GAPA was shown to interact with filamin and cortexillin I, but not cortexillin II [36]. Both filamin and cortexillin are actin cross-linking proteins, and their interaction with GAPA maps to the actin binding domain (ABD) [36]. The data suggest that GAPA interactions with filamin and cortexillin I regulate actin dynamics during cytokinesis. Therefore, although GAPA lacks the actin-binding domain found in other family members (Figure 1), it may maintain a conserved role in regulating F-actin organization through interaction with other actin binding proteins.

## 4. Mammalian IQGAPs

Mammalian cells contain three IQGAPs, IQGAP1, IQGAP2, and IQGAP3. They share a high degree of sequence homology and a similar domain structure, but differ in tissue distribution (Figure 1) [5]. IQGAP1 is ubiquitously expressed while IQGAP2 is found in liver, prostate, kidney, thryroid, stomach, platelets, salivary glands, and testis, where a unique splice variant is found, and IQGAP3 is expressed only in proliferating cells [5].

 IQGAP1 is the best studied of the three mammalian IQGAPs. Well-known functions of IQGAP1 are in actin-mediated process downstream of GTP bound Rac1 and Cdc42 including polarity, cell-cell adhesion, phagocytosis, and motility [37–39]. In addition, IQGAP1 functions as a signaling scaffold for the mitogen-activated protein kinase (MAPK) cascade [5, 40, 41]. IQGAP1 has also recently been shown to play a role in the pathogenesis of microbes including swine fever virus, *Salmonella, Pseudomonas*, and *E. coli* [42]. Changes in expression of IQGAP1 have been linked to human cancers, and many IQGAP1 binding partners also have functions in cancer progression (reviewed in [1, 5]).

 A 2006 review of IQGAP1 included a table listing 35 IQGAP1-interacting proteins [40]. Since then, at least a dozen additional binding partners have been identified from mammalian cells. Previously unrecognized functions in cell cycle progression, cytokinesis, and capping of actin filaments have recently been described, as well as new roles of IQGAP1 in differentiated cells. In the following sections, newly described IQGAP1 protein-protein interactions and the recently elucidated physiological roles of IQGAP1 in mammalian cells will be highlighted. The regulation of IQGAP2 by phosphorylation and role of IQGAP3 in proliferation will also be discussed.

## 5. IQGAP1

### 5.1. Nuclear Localization and Function in Cell Cycle Progression

A new study confirmed previously reported nuclear localization of IQGAP1 by confocal microscopy and cell fractionation in several mouse and rat cell lines [43]. Nuclear localization of IQGAP1 was cell cycle regulated, as it was enhanced by arrest at the G1/S boundary and observed during the G1/S transition in asynchronous cells [43]. IQGAP1 was previously shown to increase both *β*-catenin levels and transcriptional activity in the nucleus, but *β*-catenin is not required for IQGAP1 nuclear localization [43–45]. Inhibition of glycogen synthase kinase (GSK)-3*β* increased the nuclear levels of IQGAP1, suggesting that this signaling pathway normally regulates IQGAP1 subcellular localization [43]. Whether this regulation occurs by increased import, decreased export, or association with nuclear factors remains to be determined. Although siRNA knock-down of IQGAP1 did not change cell cycle distribution of asynchronous cells, it did lead to a delay in recovery from G1/S arrest induced by thymidine [43]. This specific role of IQGAP in cell cycle progression may be mediated through its interaction with DNA replication proteins RPA32 and PCNA [43]. Expression of a C-terminal region of IQGAP1 containing the GRD and RGCt regions in NIH3T3 cells caused an increase in cell proliferation, as well as transformation phenotypes of growth in low serum and soft agar [46]. Whether or not the promotion of cell division is due to binding of this region of IQGAP1 to RPA32 and PCNA remains to be determined.

### 5.2. Cytokinesis

Although IQGAP family members have been shown to be required for cytokinesis in *Dictyostelium, *sea urchin, and yeast cells, a role of mammalian IQGAPs in cytokinesis was uncertain [32]. However, several recent studies provide hints that this role of IQGAPs may be conserved. A study of mouse oocyte and preimplantation embryos showed that IQGAP1 was localized to cleavage furrows, and disruption of Cdc42 function using Toxin B led to disruption of IQGAP localization and cytokinesis failure [47]. In HeLa cells, expression of a truncated IQGAP1 containing the IR and WW repeats caused a large increase in the number of cells with three or more nuclei, indicating that cytokinesis may be impaired [46]. A proteomic study of mammalian cytokinesis proteins using Chinese hamster ovary (CHO) cells identified IQGAP as a midbody component [48]. The midbody is a microtubule containing structure that connects mammalian cells after contraction of the cytokinetic ring. The localization of IQGAP1 in the midbody was confirmed by immunofluorescence [48]. However, it is not clear whether IQGAP1 functions at the midbody or is a component of the cytokinetic ring or mitotic spindle that becomes concentrated at the midbody during furrowing. RNAi of the *C. elegans* IQGAP1 homolog (F09C3.1 or *pes-7*) resulted in germline defects and failure of meiotic divisions, indicating that IQGAP1 is involved in cytokinesis in nematodes [48]. The function of IQGAP1 in *C. elegans* cytokinesis has not been investigated further. Resolution of the midbody and final separation of the daughter cells occurs by a process called abscission, which requires vesicle trafficking and secretion and membrane fission at the midbody [49]. Depletion of the endosomal sorting complex required for transport I (ESCRT-I) subunit TSG101 was shown to inhibit abscission in HeLa cells, and TSG101 interacts with IQGAP1 by yeast two-hybrid [50]. These data suggest that IQGAP1 may function at the midbody during cytokinesis in human cells. Ironically, although the first functional role discovered for IQGAPs was in cytokinesis, the involvement of IQGAPs in mammalian cytokinesis is not well characterized [32]. It will be interesting to determine if the role of IQGAPs in cytokinesis is universal.

### 5.3. Exocytosis

Investigation of polarized secretion in pancreatic *β*-cell lines revealed a function for IQGAP1 in exocytosis [39]. IQGAP1, but not IQGAP2 or IQGAP3, interacted with the exocyst component EXO70 and the septin SEPT2 by coimmunoprecipitation [39]. Surprisingly, activated Cdc42 (Cdc42Q61L) inhibited, rather than enhanced, the interactions [39]. The IR and WW regions of IQGAP1 required for the interaction with EXO70 and SEPT2 [39]. IQGAP1 and EXO70 colocalize at the leading edge, in a perinuclear meshwork, and in tubular structures that may correspond to the ER [39]. Knock-down of IQGAP1 levels by RNAi leads to a reduction in EXO70 localization and a 45% decrease in insulin secretion [39]. The authors propose a regulatory role for IQGAP1 in secretion, whereby exocytosis in response to glucose occurs by Ca^2+^ regulated binding of IQGAP1 to EXO70, and activation of Cdc42 in response to growth factors inhibits interaction of IQGAP1 with EXO70 and secretion [39].

### 5.4. F-Actin Barbed End Capping

IQGAP1 has long been recognized to bind and bundle F-actin via the CHD in the N-terminus. Surprisingly, a bacterially expressed C-terminal half of IQGAP1 was shown to cap the barbed end (also known as the plus end, where most polymerization and depolymerization occurs) of F-actin filaments. Both calcium-bound calmodulin (Ca-CaM) and calcium-free calmodulin (apo-CaM) inhibited the capping activity [51]. Therefore, this work identified a new binding site for F-actin in IQGAP1 that does not resemble known capping proteins at the sequence level and a novel mechanism by which IQGAP1 affects the formation of actin structures. Previously, IQGAP1 was reported to stimulate actin nucleation through activation of N-WASp, a regulator of the Arp2/3 complex [37, 52]. In the current study the authors hypothesize that IQGAP1 functions in forming the branched actin structures found in lamellipodia through its capping activity rather than N-WASp activation [51]. Whether IQGAP1 functions in lamellapodia formation through interaction with N-WASp and/or by the barbed end capping activity will be important to determine.

### 5.5. MAPK Signaling

The best-studied mitogen activated protein kinase (MAPK) pathway is the extracellular signal-regulated kinase (ERK) cascade, which transmits signal from Raf (MAP kinase kinase kinase) to MEK (MAP kinase kinase) to ERK (MAP kinase). IQGAP1 is a scaffold for MAPK signaling, binding to B-Raf, MEK1/2, and ERK1/2, and regulating their activity in response to epidermal growth factor (EGF) (reviewed in [41]). Recently, IQGAP1 has also been shown to bind and colocalize with the EGF receptor (EGFR) [53]. Interestingly, IQGAP1 coimmunoprecipitates with EGFR independently of EGF, and therefore the interaction is unlikely to depend on receptor dimerization or phosphorylation [53]. The interaction between IQGAP1 and EGFR is abrogated by addition of Ca^2+^, suggesting that this interaction, like many others, may be inhibited by Ca-CaM [53]. The data also show that EGF promotes serine phosphorylation of IQGAP1 by protein kinase C (PKC), and that this phosphorylation enhances EGFR tyrosine phosphorylation [53, 54]. This suggests a positive feedback loop downstream of PKC acting through IQGAP1 to fully activate EGFR. IQGAP1 was also recently shown to bind the related receptor human epidermal growth factor type-2 (HER2) [55]. Importantly, IQGAP1 is required for HER2-mediated proliferation, and may therefore be a therapeutic target for HER2+ breast cancer [55].

 IQGAP1 has also been implicated in angiogenesis, or growth of new blood vessels, through its binding to the vascular endothelial growth factor receptor type 2 (VEGFR2), and two recent papers showed a role for IQGAP1 in endothelial cell proliferation and macrophage migration during neovascularization [56, 57]. Knockdown of IQGAP1 by siRNA reduced B-Raf activation and endothelial cell proliferation in response to VEGF [56]. The *in vivo* importance of IQGAP1 on angiogenesis was shown by the requirement for IQGAP1 in the chicken chorioallantoic membrane assay, as well as leg injury in IQGAP null mice [56, 58]. In the mouse model, mice lacking IQGAP1 had reduced capillary density, macrophage infiltration, and reactive oxygen species (ROS) production at the injury site [58].

 In addition to MAPK signaling downstream of receptor tyrosine kinases (RTKs), IQGAP1 may regulate signaling from the two main glutamate receptors in the central nervous system, *α*-amino-3-hydroxy-5-methyl-4-isoxazole propionic acid (AMPA) receptor, and N-methyl-D-aspartate (NMDA) receptor. IQGAP1 was previously shown to interact with a subunit of the AMPA receptor, which is involved in synaptic plasticity [59]. A new study showed that IQGAP1 interacts with the NR2A subunit and PSD-95 scaffolding protein of the NMDA receptor (NMDAR) [60]. IQGAP1-null mice lack ERK1/2 activation in response to NMDAR stimulation or fear conditioning [60]. This could be due to a lack of NMDAR at the plasma membrane due to IQGAP1's function in secretion and/or lack of IQGAP1 scaffolding activity to mediate signaling downstream of the receptor [39, 60]. Mice lacking IQGAP1 had defects in memory formation and long-term potentiation after mild stimuli [60]. In addition, the hippocampal neurons had lower dendritic spine density [60]. As decreases in spine density accompany psychiatric disorders such as schizophrenia and depression, the function of IQGAP1 in this process is of considerable interest.

 IQGAP1 was previously shown to interact with the Rap1a and Rap1b GTPases through its IQ motifs [61]. A recent report suggests that this interaction may have functional consequence in natural killer (NK) cells [62]. In stimulated NK cells, Rap1b and IQGAP1 colocalize, and active B-Raf, C-Raf, and ERK1/2 were reduced in Rap1b null cells [62]. This suggests that IQGAP1 may activate MAPK signaling downstream of Rap1b in NK cells. However, since IQGAP1, but not Rap1b, is required for NK cytotoxicity, IQGAP1 must play additional roles in NK cells [62, 63].

 Sbroggiò et al. showed that IQGAP1 is expressed in cardiomyocytes and cardiac fibroblasts [64]. IQGAP1-null mice have increased apoptosis of cardiac cells after pressure overload [64]. In these cells, IQGAP1 interacts with C-Raf rather than B-Raf, and is required for a second wave of MEK1/2 and ERK1/2 activation after stress to the organ caused by aortic banding [64]. This study suggests that IQGAP1 may act as a scaffold for B-Raf signaling in some cells and C-Raf in others.

### 5.6. Interaction with Adaptor Protein SchA

The adaptor protein SchA binds activated receptors and recruits Grb2 to activate Ras and MAPK signaling. IQGAP1 was identified as a SchA binding partner, and both are recruited to membrane ruffles after activation of the rat HER2 receptor independently of Grb2 [65]. The interacting region of IQGAP1 was mapped to a part of the IR region between amino acids 401–533 [65]. The binding of IQGAP1 to SchA occurred with and without Ca^2+^, unlike the interaction of IQGAP1 with many other proteins that is negatively regulated by Ca-CaM [65]. It is unclear whether the function of the IQGAP1 interaction with SchA is to mediate MAPK activation or to induce cytoskeletal rearrangements in response to receptor activation.

### 5.7. IQGAP1 Tyrosine Phosphorylation

As summarized above, IQGAP1 functions downstream of several RTK (receptor tyrosine kinases), and binds directly to some of them. Accordingly, there have been several studies reporting tyrosine phosphorylation of IQGAP1 after RTK activation. Proteomic studies of EGF and PDGF stimulated signaling pathways identified IQGAP after affinity purification with antiphosphotyrosine (anti-pTyr) antibodies and mass spectrometry analysis [66, 67]. IQGAP1 reacted with anti-pTyr antibody after stimulation of VEGFR2 in a c-Src dependent manner [56, 57]. Expression of ShcA was reported to increase basal phosphotyrosine phosphorylation of IQGAP1 downstream of the ErbB2/HER2 receptor [65]. S100P expression reduced anti-pTyr recognition of IQGAP1 after EGF treatment [68]. Therefore, the tyrosine phosphorylation of IQGAP1 is reportedly regulated by interacting proteins c-Src, SchA, and S100P. However, a recent report stated that no tyrosine phosphorylation of IQGAP1 was detected after EGF stimulation [53]. These authors suggested that the protein recognized previously by anti-pTyr antibody was not IQGAP1, but a co-migrating binding partner. Whether or not IQGAP1 is normally regulated by tyrosine phosphorylation remains an open question, as no tyrosine phosphorylation sites in IQGAP1 have yet been mapped and mutated to show *in vivo* function. Several recent proteomic studies have examined phosphotyrosine in cancer cells using mass spectrometry. One study found phosphorylation of IQGAP1 at Y654 and Y1510 in several nonsmall cell lung cancer (NSCLC) lines, while another found phosphorylation of Y1114 in a different NSCLC cell line [69, 70]. Phosphorylation of Y172 and Y1510 was observed in mouse NIH/3T3 cells expressing constitutively active c-Src [70, 71]. In a study of mouse fibroblasts, IQGAP1 was phosphorylated at Y1510 in nontransformed, and at Y654 and Y1510 in Src transformed fibroblasts [72]. Since IQGAP1 has been implicated in tumor metastasis, it will be important to determine if tyrosine phosphorylation of IQGAP1 in cancer cells has functional consequences or is a result of overactive tyrosine kinases.

### 5.8. mTOR and AKT Signaling

RTKs such as EGFR activate not only MAPK signaling downstream of Ras, but also protein kinase B (Akt) and mammalian target of rapamycin (mTOR) activation downstream of phosphatidylinositol 3-kinase (PI3K). Recently, IQGAP1 was shown to bind mTOR through a region containing the IR and WW repeats [46]. IQGAP1 was also shown to interact with AKT, the downstream target of mTOR, suggesting that IQGAP1 may function as a scaffold for this signaling pathway [73]. A mutant IQGAP1 that cannot interact with Rac1 or Cdc42 also does not coimmunoprecipitate mTOR or Akt, suggesting that activation of this pathway occurs downstream of the active GTPases [46, 73]. IQGAP1 was also shown to bind Akt in mouse heart extracts, and IQGAP-null mice lack activated Akt at four days after aortic banding, showing a tissue-specific function for this interaction [64].

 In addition to facilitating mTOR phosphorylation of Akt, IQGAP1 interaction with mTOR may modulate its effects on the cytoskeleton. mTOR phosphorylation of microtubule plus end binding (+TIP) protein CLIP-170 is required for the interaction of CLIP-170 and IQGAP1 in neurons [74]. Knockdown of either CLIP-170 or IQGAP1 reduced the number of neuronal dendrites, which could be rescued by jasplakinolide-forced F-actin stabilization [74]. Therefore, these data suggest that mTOR signaling in neurons coordinates microtubule and actin cytoskeletons by promoting the CLIP-170-IQGAP1 interaction.

### 5.9. Interaction with Microtubule-Binding Protein CLASP2

CLASPs are CLIP-associating proteins that track the plus ends of microtubules (+TIPs) and control microtubule dynamics. IQGAP1 had previously been linked to the microtubule cytoskeleton through its interaction with another +TIP, CLIP-170 (see above) [75]. Recently, the RGCt domain of IQGAP1 was shown to bind another +TIP, CLASP2 [76]. The interaction is negatively regulated by GSK-3*β* phosphorylation of CLASP2 [76]. The binding of IQGAP1 to CLASP2 is important for cell migration, as siRNA of either led to migration defects, and these defects could be rescued by full-length IQGAP1, but not IQGAP1 lacking the binding region for CLASP2 [76].

### 5.10. Interaction with EF Hand Protein S100P

The IQ motifs of IQGAP family members interact with calcium binding EF-hand proteins, such as CaM, myosin light chain, and S100B. Recently, IQGAP1 was identified as a Ca^2+^ dependent binding partner for S100P [68]. There are twenty-five S100 family proteins in humans, many of which are tissue-specific. First identified in placenta, S100P levels are elevated in breast, pancreas, lung, and ovary carcinomas [77]. The interaction of IQGAP1 with S100P in the presence of Ca^2+^ negatively regulated the interaction of IQGAP1 with B-Raf without affecting the binding of IQGAP1 to Cdc42 or Rac1 [68]. This selective regulation of IQGAP1 is unique to S100P, as Ca-CaM negatively regulates all known IQGAP1 interactions.

### 5.11. Structural Studies

The NMR structure of human IQGAP1 actin binding domain (ABD), which contains the CHD and flanking sequences, was recently solved and shown to be similar to the crystal structure of *S. pombe* Rng2 [78, 79]. IQGAPs have a type 3 CH family domain, containing a single rather than tandem CHD. There are two putative ABDs within the single IQGAP1 CHD, and the NMR structure suggests that actin binding may be regulated by an intramolecular interaction between an extension region that wraps around to contact the ABD [78].

 Mutational analysis of IQGAP1's interaction with Rac1 and Cdc42 showed that although the two GTPases are 71% identical, they have only partially overlapping binding sites on IQGAP1 [80]. The crystal structure for the IQGAP1 GRD has been solved, and the overall structure is similar to GAP domains from Ras GAPs [81]. Together, the studies suggest that although IQGAP1 functions as an effector, stabilizing the GTP-bound states of Cdc42 and Rac1 rather than catalyzing GTP hydrolysis, the binding of IQGAP to its target GTPases resembles binding of GAPs rather than effectors [80, 81]. All metazoan IQGAP family members have a conserved Thr1046 equivalent, which is predicted to sterically clash with the phosphate binding loop of the GTPase, disrupting the geometry for GTP hydrolysis [81]. Therefore this conserved residue is likely the structural key to the lack of GAP activity in the IQGAP GRD.

## 6. IQGAP2

IQGAP2 was recently identified as a binding partner for the protein kinase A-anchoring protein (AKAP) AKAP220 [82]. AKAPs bind cAMP-dependent protein kinases (PKA) and position them near substrates. IQGAP2 forms a ternary complex with AKAP220 and PKA, and is a substrate for PKA phosphorylation [82]. *In vitro* kinase assays showed that Thr-716, which is within the IQ motifs, is the preferred site, and interestingly IQGAP1 and IQGAP3 lack an equivalent PKA consensus site [82]. The phosphorylation of IQGAP2 at Thr-716 enhances its interaction with Rac1, but not Cdc42 [82]. *In vivo*, expression of a phosphomimetic mutation increased the number of membrane ruffles, while the nonphosphorylatable mutant had no effect [82]. It is surprising that increasing Ca^2+^ levels using an ionophore enhanced the binding of IQGAP2 to AKAP220, since most IQGAP1 protein-protein interactions are disrupted by Ca^2+^ [82]. Therefore, this study suggests that IQGAP2 is regulated by PKA and Ca^2+^ differently than IQGAP1.

## 7. IQGAP3

Mouse IQGAP3 is 57% identical to IQGAP1, yet it is the only IQGAP that interacts with active Ras as well as Cdc42 and Rac1 [83]. Surprisingly, the interaction with Ras did not depend on the GRD of IQGAP3, as fragments lacking this domain could still interact, although more weakly [83]. IQGAP3 is expressed in proliferating, Ki-67 positive cells in the small intestine and liver [83, 84]. Knockdown of IQGAP3, but not IQGAP1, in cultured cells using short interfering hairpin RNAs markedly reduced proliferation, active Ras, and active ERK2 [83]. Therefore, IQGAP3 may regulate proliferation by binding to the GTP-bound form of Ras to allow ERK activation.

## 8. Conclusions

### 8.1. Conservation of IQGAP Functions

The most conserved function of IQGAP family members is their role as a scaffold to link multiple proteins together. However, the biological processes facilitated by IQGAP's scaffolding may differ between cells. For example, the scaffolding function of Rng2 is important in recruiting proteins to the nodes for regulated assembly of the actomyosin ring in fission yeast, and the scaffolding function of IQGAP1 is necessary for MAPK signal transduction in migration and angiogenesis. It may be that IQGAPs are important for F-actin regulation downstream of Ras family GTPases for processes such as cell division, motility, and exocytosis in all eukaryotic cells, and that a role for IQGAPs in receptor mediated signaling pathways evolved later.

 In all yeast species studied, there is a single IQGAP family member that plays an essential function in cytokinesis by recruiting F-actin to the contractile ring. In *Dictyostelium*, two of the three characterized IQGAPs have overlapping functions in cytokinesis, as deletion of both *GAPA* and *GAP1* leads to a complete failure of cytokinesis while single deletions are impaired to a lesser extent [32]. However, it is unclear whether IQGAPs are required for cytokinesis in mammalian cells. Recent studies suggest that IQGAP1 localizes to the cleavage furrow or midbody, regions associated with cytokinesis, in some cell types [47, 48]. Association of IQGAP1 with a member of the ESCRT complex suggests that IQGAPs in mammalian cells may function in abscission, the final stage of cytokinesis, rather than in the initial recruitment of F-actin to the cytokinetic ring as in lower eukaryotes. Further studies are needed to elucidate the function of IQGAPs in mammalian cytokinesis.

 IQGAPs have long been known to bind and bundle actin filaments. New studies suggest that IQGAPs may also function to stimulate actin polymerization via nucleation or barbed end capping. Studies in fission yeast showed that its IQGAP, Rng2, can nucleate *in vitro* filament assembly as well as bundle actin filaments via the Rng2Ns region (CHD and additional amino acids). Barbed end binding of Rng2Ns was not observed, perhaps because this activity requires the GRD [14]. In contrast, full-length IQGAP1 alone did not stimulate actin assembly *in vitro*, but did increase Arp2/3 mediated nucleation in the presence of N-WASP [37]. Recently, the GRD of IQGAP1 was shown to bind to the barbed end of F-actin, which may be important for its role in promoting nucleation of branched filaments via Arp2/3 [51]. Therefore, evidence suggests that IQGAPs in both fission yeast and mammalian cells function to stimulate actin nucleation. However, either this is accomplished through two different mechanisms, or *in vitro* assays do not give a true picture of how IQGAPs act in the cell to promote F-actin assembly.

 Another conserved function of IQGAPs is in polarized secretion. In budding yeast, Iqg1 interacts with the exocyst component Sec3 and the spatial marker Bub4 to target exocytosis to the bud neck [27]. Polarized secretion is important in budding yeast for targeting growth to the bud and for deposition of cell wall during and after cytokinesis. In mammalian cells, polarization of cell growth and secretion is important during migration and differentiation. IQGAP1 plays a role in regulating secretion through its interaction with the exocyst component EXO70 and the septin SEPT2 [39]. Therefore, the interaction between IQGAP and the exocyst is conserved in budding yeast and mouse cells.

 IQGAPs function in cell motility, playing a role in *Dicty* chemotaxis and mammalian lamellapodia formation. Chemotaxis in *Dictyostelium* occurs via Ras activation and signaling through PI3K and Akt. IQGAP/cortexillin complexes are important for correct polarization of cells in response to the signal [33]. Activation of FGFR1 and VEGFR leads to IQGAP1 dependent cell motility in mammalian cells, which also involves Akt [37, 57]. Many details of these processes remain to be resolved, such as whether the leading edge in *Dicty* has branched actin structures similar to those in lamellapodia, and whether there are other similarities besides the involvement of Akt in the signaling pathways involved in *Dicty* chemotaxis and growth factor stimulated mammalian cell motility.

 Although promotion of cell proliferation is a function shared by IQGAP1 and IQGAP3, it is mediated by different mechanisms. IQGAP3 binds H-Ras directly and mediates proliferation thorough ERK activation while IQGAP1 effects on proliferation appear to act through Cdc42 and mTOR [46, 83]. Unlike IQGAP3, IQGAP1 does not bind H-Ras or R-Ras. IQGAP1 was identified in a proteomic screen as interacting with M-Ras, although no function of this interaction has been described [85]. It may be that different IQGAPs bind different Ras subfamily members. Further tissue-specific studies are needed to examine the different roles of IQGAP1, 2, and 3 in differentiated and proliferating cells.

### 8.2. Conservation of IQGAP Binding Partners

IQGAP1 interacts with an astounding number of proteins (Table 1). As the number of binding partners continues to rise, it will be important for the field to sort out how the interaction of IQGAP1 with one protein influences its activity or binding to other molecules. For example, it has been proposed that binding of Ca-CaM to IQGAP1 induces a closed conformation that prevents binding to other proteins, but since so many protein-protein interactions require the IQ domains, competitive binding is another mechanism that may regulate the interactions.

 Interaction of IQGAPs with small GTPases is common among all family members, and the interaction with F-actin occurs through the CHD in yeasts and mammals while the interaction of Dicty IQGAPs may be mediated through actin binding proteins such as cortexillin. Although there is diversity in binding partners, there are some interactions that appear to be conserved (Table 1).

 One conserved interaction may be between IQGAPs and formin proteins. Formins are actin nucleating proteins involved in cytokinesis, polarization, and migration. Mammalian IQGAP1 is required for localization of the formin Dia1 in migrating cells, and CaIqg1 coimmunoprecipitates with the formins Bni1 and Bnr1 (Table 1) [3, 28]. In *S. pombe*, there is a genetic interaction between Rng2 and the formin Cdc12 [6]. In *S. cerevisiae*, both formins and Iqg1 are required for actin ring formation, suggesting that the interaction may also be conserved in these cells [86]. The function of the interaction between IQGAP and formin may be to regulate formin localization, activation, or stimulate polymerization activity [87].

### 8.3. Regulation of IQGAPs by Phosphorylation

While regulation of IQGAP family members by phosphorylation is a common mechanism in many different cells, the modified residues and responsible kinases may not be well conserved.

 In yeast cells, the activity of IQGAP must be cell-cycle regulated to ensure spatiotemporal regulation of actomyosin ring assembly. While both CaIqg1 and ScIqg1 protein levels peak in mitosis, Rng2 levels are constant throughout the cell cycle [3, 25, 88]. CaIqg1 and ScIqg1 have multiple conserved CDK sites in the N-terminus, but Rng2 shares only one (Figure 2) [3]. Mass spectrometry analysis of *S. pombe* identified a single phosphorylation site in Rng2, but the function of this phosphorylation and kinase responsible are unknown (Figure 2) [89]. Therefore, regulation of yeast IQGAPs during the cell cycle may depend on CDK in *C. albicans* and *S. cerevisiae*, but occurs through an unidentified mechanism in *S. pombe*. A large-scale analysis of mitotic phosphorylation in human cells identified phosphorylation of Ser-330 in IQGAP1 specifically during M phase [90]. This serine is in a CDK consensus site, and further experiments are needed to determine if IQGAP1 is a bona fide CDK target. The relative levels of IQGAP1 in G1 and M phase arrested cells were similar, so human IQGAP1 protein levels may not be cell cycle regulated [90].

 The three human IQGAPs are highly conserved, and may be regulated similarly by PKC but differently by PKA. IQGAP1 is thought to be negatively regulated through an intramolecular interaction in the C-terminus that is relieved upon phosphorylation of Ser-1443 by protein kinase C (PKC) *ε* [91]. Phosphorylation of IQGAP1 by PKC facilitates its binding to Cdc42. The serine equivalent of 1443 is conserved in IQGAP2 (Ser-1358) and IQGAP3 (Ser-1424), and phosphorylation of these sites has been observed in proteomic studies [92]. The functional consequences of phosphorylating the Ser-1443 equivalent in IQGAP2 and IQGAP3 are unknown. IQGAP2 is phosphorylated by PKA at Thr-716, which increases its interaction with Rac1 [82]. The Thr at 716 is not conserved between mammalian IQGAPs. Therefore, phosphorylation of IQGAPs in mammalian cells may have a similar effect of promoting the interaction with GTPases, and IQGAP2 may be regulated by PKA in addition to PKC.

 In conclusion, the study of IQGAPs in many different organisms has led to the discovery of a wide range of associations and functions. The challenge for the future is to ascertain how IQGAP interacts with particular subsets of its binding partners to differentially regulate cellular processes.

## Figures and Tables

**Table 1:** Binding partners of IQGAPs. Binding partners of IQGAP family members are grouped by function and shown under the domain required for interaction. Proteins that are known to interact, but the IQGAP domain required has not been determined are listed under unmapped interactions. Proteins in italics represent genetic interactions, while all others are physical interactions. To draw attention to similar binding partners, the exocyst components are highlighted purple, formins are highlighted bright green, myosin light chains are shown in dark green, and calmodulins are shown in yellow. F-actin highlighted in red.